# The integrin αvβ6/TGF-β1/Smad4 signaling pathway regulates ENaC in alcohol induced ARDS

**DOI:** 10.3389/fphar.2026.1931568

**Published:** 2026-10-16

**Authors:** Xue Dai, Xue-Ling Liu, Mei-Jun Liu, Ting Li, Dao-Xin Wang, Wang Deng

**Affiliations:** 1 Department of Respiratory and Critical Care Medicine, The Second Affiliated Hospital of Chongqing Medical University, Chongqing, China; 2 Key Laboratory of Respiratory Inflammatory Injury and Precision Diagnosis and Treatment, Chongqing Municipal Health and Health Committee, Chongqing, China; 3 Chongqing Key Laboratory of Precision Medicine and Prevention of Major Respiratory Diseases, Chongqing, China

**Keywords:** acute respiratory distress syndrome, alveolar fluid clearance, chronic alcohol abuse, epithelial sodium channel, sodium-potassium pump

## Abstract

**Introduction:**

Acute respiratory distress syndrome (ARDS) is characterized by impaired alveolar-capillary permeability and pulmonary edema. Alveolar fluid clearance (AFC) is strongly associated with patient prognosis. This study aimed to investigate whether chronic alcohol consumption affects sodium channel regulation in ARDS via the Integrin αvβ6/TGF-β1/Smad4 signaling pathway.

**Methods:**

We established chronic alcohol exposure models in mice and MLE-12 cells, followed by lipopolysaccharide (LPS)-induced ARDS in both systems. The Integrin αvβ6/TGF-β1/Smad4 pathway was inhibited to explore its regulatory effect on ENaC and Na^+^,K^+^-ATPase during alcohol-induced ARDS.

**Results:**

We found that chronic alcohol consumption exacerbated lung injury by activating this signaling pathway, which suppressed ENaC and Na^+^,K^+^-ATPase protein expression. Inhibition of the pathway alleviated pulmonary edema and inflammation and improved survival rates. Both in vivo and in vitro results demonstrated that the Integrin αvβ6/TGF-β1/Smad4 pathway regulates ENaC and Na^+^,K^+^-ATPase in alcohol-induced ARDS.

**Conclusion:**

These findings may clarify the regulatory mechanisms of sodium channels in ARDS associated with chronic alcohol consumption.

## 1 Introduction

Acute respiratory distress syndrome (ARDS) is a critical condition involving diffuse alveolar injury caused by various pathogens and characterized by severe hypoxemia. Pulmonary endothelial and alveolar epithelial damage disrupts the alveolar-capillary barrier, leading to edema and impaired gas exchange, ultimately causing respiratory failure (Matthay and Zemans, 2011). Over the past decade, ARDS hospital mortality rates have remained between 30% and 40% (Matthay et al., 2019). ARDS pathogenesis involves dysregulated inflammation and oxidative stress; targeting these pathways improves lung injury. Despite extensive preclinical and clinical studies involving glucocorticoids, surfactants, free radical scavengers, and β-agonists, no therapies have successfully reduced ARDS mortality in randomized controlled trials (Fan et al., 2018). Although progress has been made in understanding ARDS pathogenesis, the exact mechanisms by which specific risk factors contribute to ARDS remain unclear, warranting further research.

Chronic alcohol consumption remains a significant global public health concern and contributes to a substantial disease burden (Fan et al., 2018) (GBD, 2016 Alcohol Collaborators, 2018). While its effects on psychiatric disorders and liver disease are well recognized, alcohol-induced pulmonary injury is less understood. Chronic alcohol abuse is a major risk factor for ARDS development (Marten et al., 2020) (Fung and Hyzy, 2018). Clinical studies show that alcohol consumers with ARDS have a 3- to 4-fold higher incidence and mortality rate compared to non-drinkers, further increasing healthcare burdens (Kershaw and Guidot, 2008). Nearly 50% of ARDS cases are associated with chronic alcohol use—a condition termed “alcoholic lung”. Research also indicates that alcohol abuse worsens ARDS outcomes in COVID-19 patients (Lassen et al., 2021). However, the mechanisms by which alcohol increases ARDS risk remain largely unknown (Simou et al., 2018). Potential mechanisms include structural damage to alveolar epithelial cells, increased reactive oxygen species (ROS) production, breakdown of the epithelial barrier, and impaired alveolar fluid reabsorption (Huang et al., 2024). Understanding how alcohol abuse affects ARDS development and progression is crucial for developing targeted therapies.

Efficient alveolar edema fluid clearance is essential for restoring gas exchange and correcting hypoxemia in ARDS, making it a key therapeutic goal (Huppert and Matthay, 2017). Alveolar epithelial cells are central to maintaining the alveolar barrier and fluid balance. Transport channels such as aquaporins, chloride channels, and potassium channels, localized in alveolar epithelial cells, contribute to alveolar fluid clearance (AFC), but sodium ion (Na^+^) drive transepithelial sodium transport in alveolar epithelium, critically determining pulmonary edema resolution and playing a key role in AFC (Vadász and Sznajder, 2017) (Vadász et al., 2007). Major sodium transporters in alveolar epithelial cells include Na^+^,K^+^-ATPase and the epithelial sodium channel (ENaC) (Wynne et al., 2017). These channels facilitate directional sodium transport and help resolve pulmonary edema (Matthay et al., 2002). Understanding their regulation in alcohol-induced ARDS is vital.

Chronic alcohol use upregulates integrin αvβ6 in alveolar epithelial cells, facilitating TGF-β1 activation and weakening barrier integrity, thereby increasing susceptibility to lung injury (Curry-McCoy et al., 2013). Alcohol upregulates Integrin αvβ6 to damage the epithelial-mesenchymal integrity in colorectal cancer (Nong et al., 2022). Integrin αvβ6 is specifically expressed in epithelial cells and serves as a key activator of TGF-β1 (Shi et al., 2011). Chronic alcohol exposure exacerbates acute lung injury via TGF-β1 (Mitchell and Guidot, 2007). The underlying mechanism may involve promoting oxidative stress by reducing glutathione in alveolar epithelial cells, and TGF - β1 further suppresses glutathione synthesis, impairing surfactant function and exacerbating lung injury (Bechara et al., 2005). *In vitro* studies show that TGF-β1 accumulation in alveoli impairs epithelial barrier function (Overgaard et al., 2015). Renal epithelial cell studies demonstrate that TGF-β1 activation inhibits ENaC expression (Wakida et al., 2006). The Integrin αvβ6-mediated activation of TGF-β1 may serve as a key trigger for alcohol-induced ARDS.

TGF-β1 is a key activator of Smad4 (Zhang et al., 2018) (Du et al., 2018). TGF-β1 inhibits transcription by engaging SMAD4 in lung cancer, Smad4 is a central mediator in the TGF-β1 signaling pathway (Zhang et al., 2018). Network pharmacology and *in vivo* studies indicate that Smad4 is a potential therapeutic target for ARDS (Lu et al., 2021). Furthermore, *in vitro* studies indicate that TGF-β1 suppresses ENaC expression via Smad4 in renal collecting duct cells (Chang et al., 2008). The involvement of the Integrin αvβ6/TGF-β1/Smad4 axis in regulating sodium channels during alcohol-induced ARDS warrants further exploration.

This study aimed to investigate the role of the Integrin αvβ6/TGF-β1/Smad4 pathway in regulating inflammation and sodium channel expression in a chronic alcohol-induced ARDS model by selectively inhibiting this pathway. Consistent findings in both *in vitro* and *in vivo* models suggest that this Integrin αvβ6/TGF-β1/Smad4 pathway plays a crucial role in ARDS associated with chronic alcohol consumption.

## 2 Materials and methods

### 2.1 Animal

A cohort of male (Pommerolle et al., 2024) C57BL/6J mice (6–8 weeks old, 22–24 g body weight) was procured from Hunan SJA Laboratory Animal Co., Ltd. The animals were housed in an SPF facility at the Second Affiliated Hospital of Chongqing Medical University under controlled conditions: a 12-h light/dark cycle (8:00 a.m. to 8:00 p.m.), constant temperature (21 °C ± 1 °C), and *ad libitum* access to standard chow and water. Each group contained a minimum of three biological replicates, in line with standard rodent ARDS mechanistic study protocols. All samples were measured in triplicate to minimize detection bias, with consistent statistical differences validating result reliability.All procedures involving animals were approved by the Institutional Animal Care and Use Committee (IACUC) of Chongqing Medical University. Each mouse was sequentially numbered. Random numbers were generated via the Excel RANDBETWEEN function, and mice were sorted by these values and assigned to experimental groups to avoid selection bias. Mice were purchased within a narrow weight range (22–24 g) to minimize baseline weight variation. This randomization procedure was reported following the ARRIVE 2.0 guidelines.

### 2.2 Chronic alcohol-induced ARDS model

Mice were randomly assigned to the following groups: control, ARDS, chronic alcohol + ARDS, chronic alcohol + ARDS + GSK3008348, and chronic alcohol + ARDS + SB431542. Mice had free access to water or alcohol (Sigma, USA). The sample size per group was determined based on our preliminary data and published comparable studies to ensure adequate statistical power,the alcohol concentration was gradually increased from 5% to 20% (v/v) over the first week (5% for days 1–2, 10% for days 3–4, 15% for days 5–6, and 20% from day 7) and maintained at 20% (v/v) for 6 weeks in the chronic alcohol group (Sang et al., 2022), the mean blood alcohol concentration (BAC) in animals can reach 150–200 mg/dL or higher (Martins et al., 2023; McCaskill et al., 2012). The control group had unrestricted access to water. Body weights were not significantly affected by ethanol exposure. To induce ARDS, mice were anesthetized with sodium pentobarbital administered intraperitoneally at 50 mg/kg using sterile injection devices. LPS solution (1 mg/mL; 5 mg/kg; *Escherichia coli* serotype O55:B5,Solarbio) or PBS was administered intratracheally using a 20-gauge indwelling needle catheter. GSK3008348 (MCE; 1 mg/mL; 3 mg/kg, bid), a selective integrin αvβ6 inhibitor, was administered intranasally 1 h before LPS (John et al., 2020). SB431542 (MCE; 1 mg/mL; 5 mg/kg), a selective TGF-β1 inhibitor, was injected intraperitoneally 1 h prior to LPS administration (Bao et al., 2023). Some mice were euthanized 24 h post-LPS; the remaining were monitored daily for 7-day survival.

### 2.3 Lung wet/dry (W/D) ratio

Mice were humanely euthanized via intraperitoneal injection of an overdose of sodium pentobarbital (150 mg/kg). Lungs were harvested post-euthanasia. Wet weight was recorded immediately; dry weight was measured after oven-drying at 70 °C for 48 h. The W/D ratio was calculated.

### 2.4 BALF collection

The trachea was cannulated using a 20-gauge needle, and lungs were lavaged three times with 1 mL cold sterile PBS. Bronchoalveolar lavage fluid (BALF) was centrifuged at 1,000 × g for 10 min at 4 °C. The supernatant was used to quantify protein and cytokine levels; the pellet was reserved for cell counting. Total protein concentration was measured using a BCA kit (Beyotime, Shanghai). Cell counts were obtained with an automated cell counter (Thermo Fisher Scientific, USA).

### 2.5 ELISA

TNF-α and IL-6 levels in serum and BALF were measured using ELISA kits (Ruixinbio, Quanzhou, China), according to the manufacturer’s instructions.

### 2.6 H&E staining

Lung tissues for histological analysis were obtained from a separate cohort of mice. Tissues were fixed in 4% paraformaldehyde for 48 h, paraffin-embedded, sectioned at 5 μm, stained with H&E, and observed under a light microscope (KFBIO KF-PRO-120, Ningbo). Lung injury was semi-quantitatively scored: 0 (none), 1 (<25%), 2 (25%–50%), 3 (50%–75%), 4 (>75%). The total score ranged from 0 to 16. Two independent researchers evaluated the samples, and the average score was used (Zhai et al., 2022).

### 2.7 Evans blue staining

Mice were anesthetized as described above and injected intravenously with 2% Evans Blue (5 mL/kg) (Aladdin, China). After 2 h, they were humanely euthanized and perfused with cold PBS for 5 min. Lungs were homogenized in 1 mL formamide, incubated overnight at 4 °C, centrifuged (1,000 × g, 15 min), and supernatants were collected. Absorbance was measured at 620 nm and expressed as μg EBD/g wet lung tissue.

### 2.8 Na^+^,K^+^-ATPase activity assay

According to the manufacturer’s instructions of the Na^+^,K^+^-ATPase activity assay kit (Solarbio, Shanghai, China), the assay was performed. Briefly, prepared reagents were added to lung tissues or MLE-12 cells, followed by homogenization or ultrasonication for cell disruption. After centrifugation, the resulting supernatant was collected for subsequent measurement.

### 2.9 Western blot (WB)

A protein extraction kit was employed to isolate proteins from lung tissue samples and cultured cells. Proteins were extracted from lung tissue samples and cultured cells using a protein extraction kit (Beibo Biology, Shanghai, China), and protein concentrations were determined with a BCA protein assay kit (Beyotime, Shanghai, China) to standardize subsequent sample loading volumes.For protein electrophoresis, the lysates (30 µg per well) were first mixed with 4×SDS-PAGE loading buffer (Beyotime) and heated, followed by loading on 10% SDS-PAGE gel (Genefist, China) for separation. Proteins were transferred to membranes, blocked, and incubated with primary antibodies overnight at 4 °C. After washing, membranes were incubated with secondary antibodies at room temperature for 60 min and visualized via chemiluminescence (Biosharp, China). Band intensities were analyzed using ImageJ (NIH). Antibodies used included integrin β6 (1:2000, ZEN-BIO Inc., Chengdu, China), TGF-β1 (1:10000, Proteintech, Wuhan, China), Smad4 (1:1,000, Global Gene Biotech, Anhui, China), Na^+^,K^+^-ATPase α1 (1:200000, ABclonal, Wuhan, China), Na^+^,K^+^-ATPase β1 (1:2000, ABclonal, Wuhan, China), ENaC-α (1:2000, Global Gene Biotech, Anhui, China), ENaC-β (1:1,000, Proteintech, Wuhan, China), ENaC-γ (1:1,000, Huabio, Hangzhou, China), and secondary antibody (1:15000, ABclonal, Wuhan, China).

### 2.10 Immunofluorescence (IF)

Lung tissue sections underwent dewaxing, rehydration, and heat-induced antigen retrieval (sodium citrate buffer, pH 6.0), followed by natural cooling at room temperature,and were blocked with 5% BSA; MLE-12 cells were fixed with cold formaldehyde and blocked with goat serum. Samples were incubated overnight with primary antibodies at 4 °C, washed, then incubated with secondary antibodies at room temperature for 1 h. DAPI was used for nuclear staining. Samples were mounted and visualized using a fluorescence microscope. Antibodies were Na^+^,K^+^-ATPase α1 (1:400, ABclonal), Na^+^,K^+^-ATPase β1 (1:100, ABclonal), ENaC-α (1:100, Global Gene Biotech), ENaC-β (1:100, Proteintech), ENaC-γ (1:100, Huabio), and secondary antibody (1:200, ABclonal).

### 2.11 Cell culture and treatment

MLE-12 cells (type II alveolar epithelial cells) purchased from HyCyte (Suzhou Haixing Biotechnology, Suzhou, China, Cat.) were cultured in DMEM/F-12 with 10% FBS and 1% penicillin-streptomycin at 37 °C, 95% air, and 5% CO_2_ within a standard CO_2_ incubator. Cells were seeded in six-well plates, followed by a 24-h incubation period prior to ethanol (60 mM) exposure (Overgaard et al., 2015). After 24-h ethanol treatment, the medium was replaced, and cells were treated with LPS (10 μg/mL), PBS or ethanol. Inhibitor groups received GSK3008348 (0.1 μM) or SB431542 (10 μM). After 24 h, the cells were harvested for analysis.

For siRNA transfection, equal amounts of Lipofectamine 3000 and equal volumes of Opti-MEM (reduced-serum medium) were applied to all groups as the transfection dilution vehicle. Only the si-Smad4 group received siRNA targeting Smad4 (sense strand: 5′-GCA​AUU​GAG​AGU​UUG​GUA​ATT-3′; antisense strand: 5′-UUA​CCA​AAC​UCU​CAA​UUG​CTT-3′). Cells were incubated with the transfection mixture for 6 h. The medium was then replaced, and cells were cultured for an additional 72 h prior to analysis.

### 2.12 Scratch assay

Confluent monolayers (>95%) in six-well plates were scratched with a 200 μL pipette tip. Cells were washed with PBS, and migration was assessed 48 h later under an inverted microscope.

### 2.13 CCK8 cell viability assay

MLE-12 cells (5000/well) were seeded in 96-well plates using 100 μL culture medium, subsequent to which ethanol, LPS, GSK3008348, or SB431542 treatment was administered. After 24-h treatment with ethanol (60 mM), LPS (10 μg/mL) was added. In drug-treated groups, GSK3008348 (0.1 μM) or SB431542 (10 μM) were simultaneously administered with LPS and ethanol. After 24 h, cell viability was assessed using the CCK8 kit per manufacturer’s instructions.

### 2.14 Statistical analysis

Data normality was assessed using the Shapiro-Wilk test. Results were expressed as mean ± SD. When the normality assumption was satisfied, one-way ANOVA followed by Tukey’s HSD post-hoc test was applied. For datasets that failed the normality test, Kruskal-Wallis test with Dunn’s multiple comparison test was used instead. Survival analysis was performed with Kaplan-Meier curves. All analyses were conducted using GraphPad Prism v9.1 (San Diego, CA, USA), and p < 0.05 was considered statistically significant.

## 3 Results

### 3.1 Validation of vehicle effects and screening of alcohol concentration-time gradient

*In vivo* studies demonstrated that lung tissues from experimental groups treated with the vehicle (DMSO) or the drug (SB431542) exhibited well-preserved alveolar architecture and an absence of inflammatory cell infiltration or other pathological alterations upon HE staining, showing no significant differences compared to the control group. The semi-quantitative scores showed no statistically significant difference compared to the control group (Figure 1A). Both *in vivo* and *in vitro* studies demonstrated that while the drug group significantly inhibited TGF-β1 protein expression, the vehicle group showed no significant alteration in TGF-β1 protein levels, indicating that the vehicle does not affect TGF-β1 protein expression (Figures 1B,C). *In vitro*, CCK-8 assay showed no significant difference in cell viability between the experimental groups (treated with vehicle and drug) and the control group (Figure 1D). The findings demonstrate that the vehicle has minimal impact on experimental results *in vivo* and *in vitro*.

**FIGURE 1:**
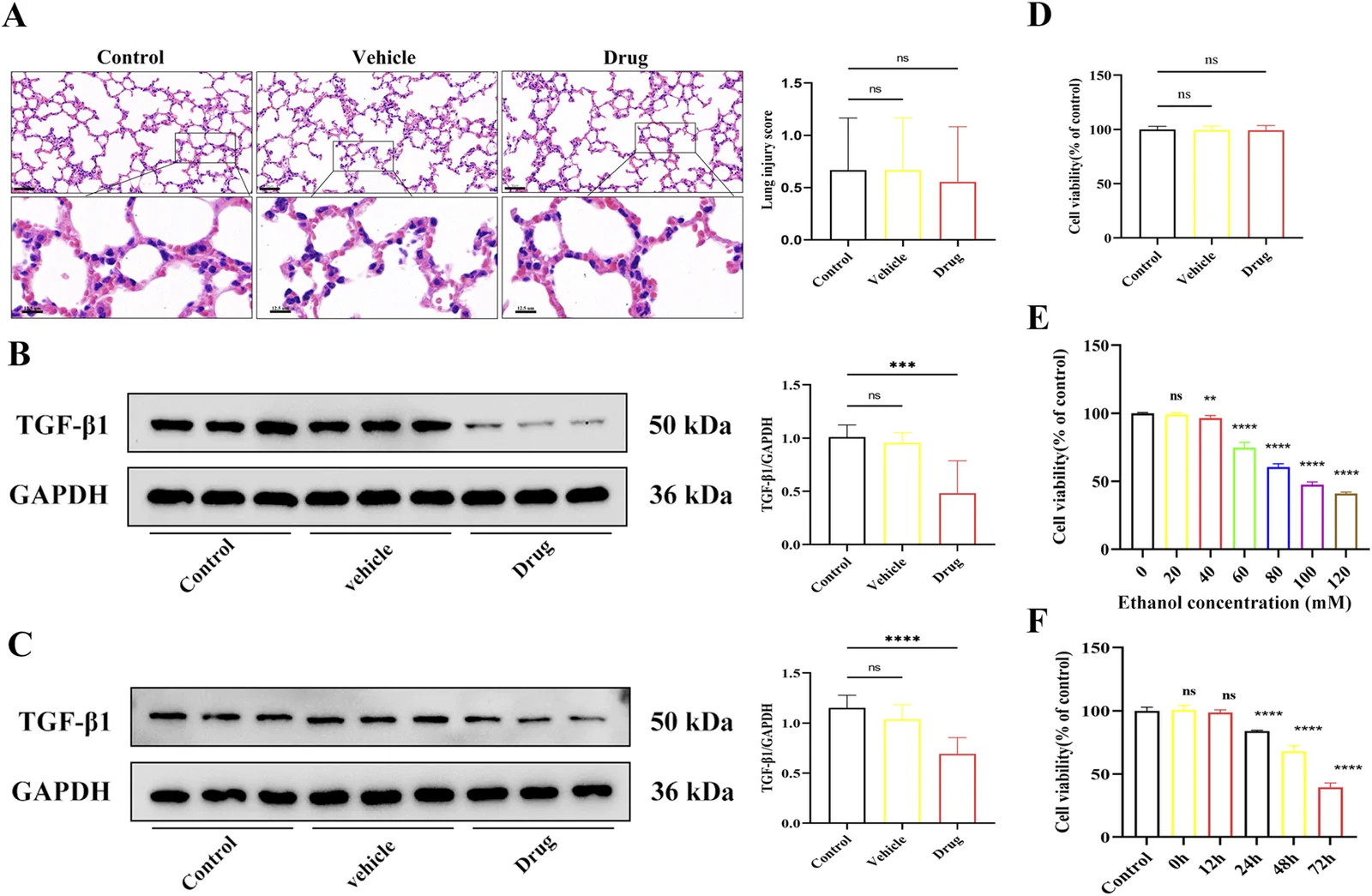
*In vivo* and *in vitro* experiments verified that DMSO had minimal impact, and *in vitro* screening determined the optimal alcohol concentration (60 mM) and exposure time (48 h). **(A)** H&E staining (scale bars, upper: 50μm, lower:12.5 μm) and lung injury score were analyzed. **(B)** Western blotting assessed TGF-β1 expression in lung tissue to evaluate the vehicle’s influence on TGF-β1 when the drug was pharmacologically active. **(C)** Western blot analysis of TGF-β1 protein expression in MLE-12 cells, and **(D)** CCK-8 assay assessing the vehicle’s effect on MLE-12 cell viability. **(E)** MLE-12 cells were treated with different alcohol concentrations (0, 20, 40, 60, 80, 100, 120 mM) for 24 h, and cell viability was assessed by CCK-8 assay. **(F)** After treatment with alcohol (60 mM), MLE-12 cells were assessed for cell viability by CCK-8 assay at different time points (0, 12, 24, 48, 72 h). Data are presented as mean ± SD (n = 3), with significance levels indicated as *P < 0.05,**P < 0.01, ***P < 0.001, and****P < 0.0001.

*In vitro* experiments were conducted to evaluate the effects of varying alcohol concentrations (0, 20, 40, 60, 80, 100, 120 mM) and exposure durations (0, 12, 24, 48, 72 h) on MLE-12 cell viability for optimal experimental condition screening.The results identified 60 mM alcohol for 48 h as the optimal condition for subsequent experiments (Figures 1E,F), effectively simulating chronic alcohol exposure effects while minimizing excessive cellular damage that could interfere with the results.

### 3.2 Integrin αvβ6/TGF-β1/Smad4 pathway mediates chronic alcohol abuse-exacerbated lung injury in ARDS mice

After successful modeling across all groups, lung tissues were harvested for H&E staining to assess the severity of pulmonary injury. Semi-quantitative scoring indicated that chronic alcohol-induced ARDS resulted in more severe injury, while inhibition of the Integrin αvβ6/TGF-β1/Smad4 pathway significantly alleviated it (Figure 2A). These results suggest that chronic alcohol abuse aggravates pulmonary injury, whereas Integrin αvβ6/TGF-β1/Smad4 pathway inhibition reduces tissue damage.Following intratracheal LPS administration and 7-day observation, the survival rates differed significantly between the chronic alcohol-induced ARDS group and the pathway-inhibited group. Compared with the non-chronic alcohol-induced ARDS group, the chronic alcohol-induced ARDS group had significantly lower survival rates. Integrin αvβ6/TGF-β1/Smad4 pathway inhibition resulted in significantly increased survival rates (Figure 2B). Vascular permeability and pulmonary edema were assessed using Evans blue dye extravasation (Figure 2C), W/D ratio (Figure 2D), and BALF total protein content (Figure 2E). Chronic alcohol exposure significantly worsened lung injury and pulmonary edema, while inhibition of Integrin αvβ6 or TGF-β1 mitigated these pathological changes. These findings demonstrate that the Integrin αvβ6/TGF-β1/Smad4 axis regulates pulmonary injury severity, vascular permeability, edema, and survival in alcohol-induced ARDS.

**FIGURE 2:**
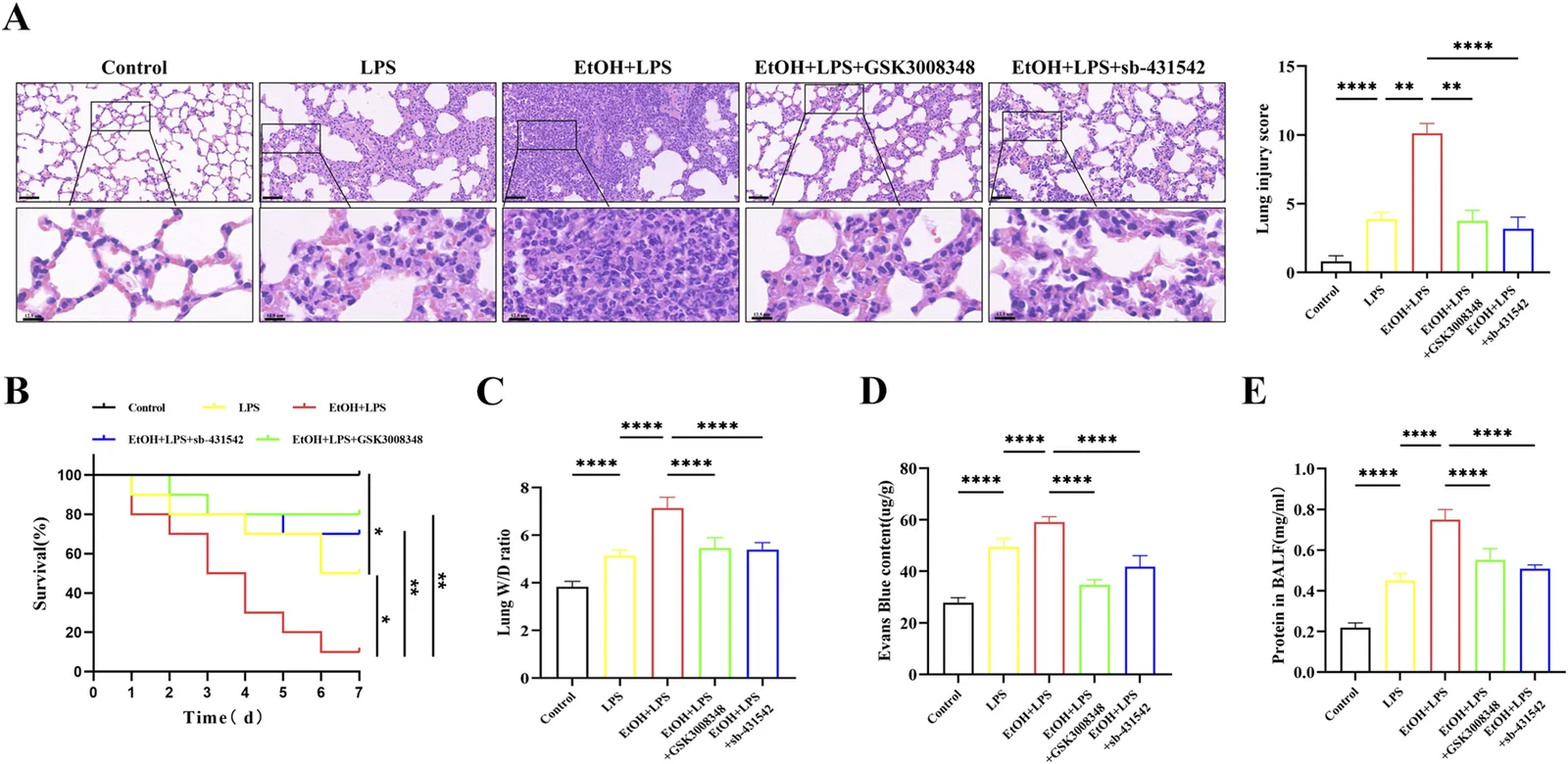
The Integrin αvβ6/TGF-β1/Smad4 pathway mediates chronic alcohol-induced ARDS progression in mice, as evidenced by exacerbated pulmonary injury, increased mortality, and worsened pulmonary edema; its inhibition alleviates these alcohol-driven pathologies. **(A)** H&Estaining (scale bars, upper: 50μm, lower:12.5 μm) and lung injury score were analyzed (n = 4). **(B)** 7-day survival rate of mice (n = 10). **(C)** Lung wet/dry weight ratio (n = 3). **(D)** Evans blue assay in mice (n = 3). **(E)** Total protein level in BALF (n = 3). Data are presented as mean ± SD, with significance levels indicated as *P < 0.05, **P < 0.01, ***P < 0.001, and ****P < 0.0001.

### 3.3 The integrin αvβ6/TGF-β1/Smad4 pathway mediates chronic alcohol-induced ARDS-associated inflammation and cellular dysfunction

The Integrin αvβ6/TGF-β1/Smad4 signaling pathway plays a key role in chronic alcohol-induced ARDS. TNF-α (Figure 3A) and IL-6 (Figure 3B) in BALF were measured by ELISA, TNF-α (Figure 3C) and IL-6 (Figure 3D) in serum were also measured by ELISA, which revealed that chronic alcohol-induced ARDS exhibited significantly elevated inflammatory cytokine levels, which were reversed by pathway inhibition. Total cell counts in BALF revealed significant differences between the chronic alcohol-induced ARDS group and the pathway-inhibited group (Figure 3E). *In vitro* studies showed that the chronic alcohol-induced ARDS group exhibited significantly higher cytotoxicity than the ARDS group, which was ameliorated by pathway inhibition (Figure 3F). Additionally, alveolar epithelial cell migration evaluated using scratch assays revealed significantly suppressed cell migration in the chronic alcohol-induced ARDS compared with the ARDS group, an effect that was effectively ameliorated through targeted pathway intervention (Figure 3G). Experimental studies demonstrate that inhibiting Integrin αvβ6/TGF-β1/Smad4 pathway alleviates the inflammatory response and attenuates cytotoxicity induced by chronic alcohol consumption in ARDS.

**FIGURE 3:**
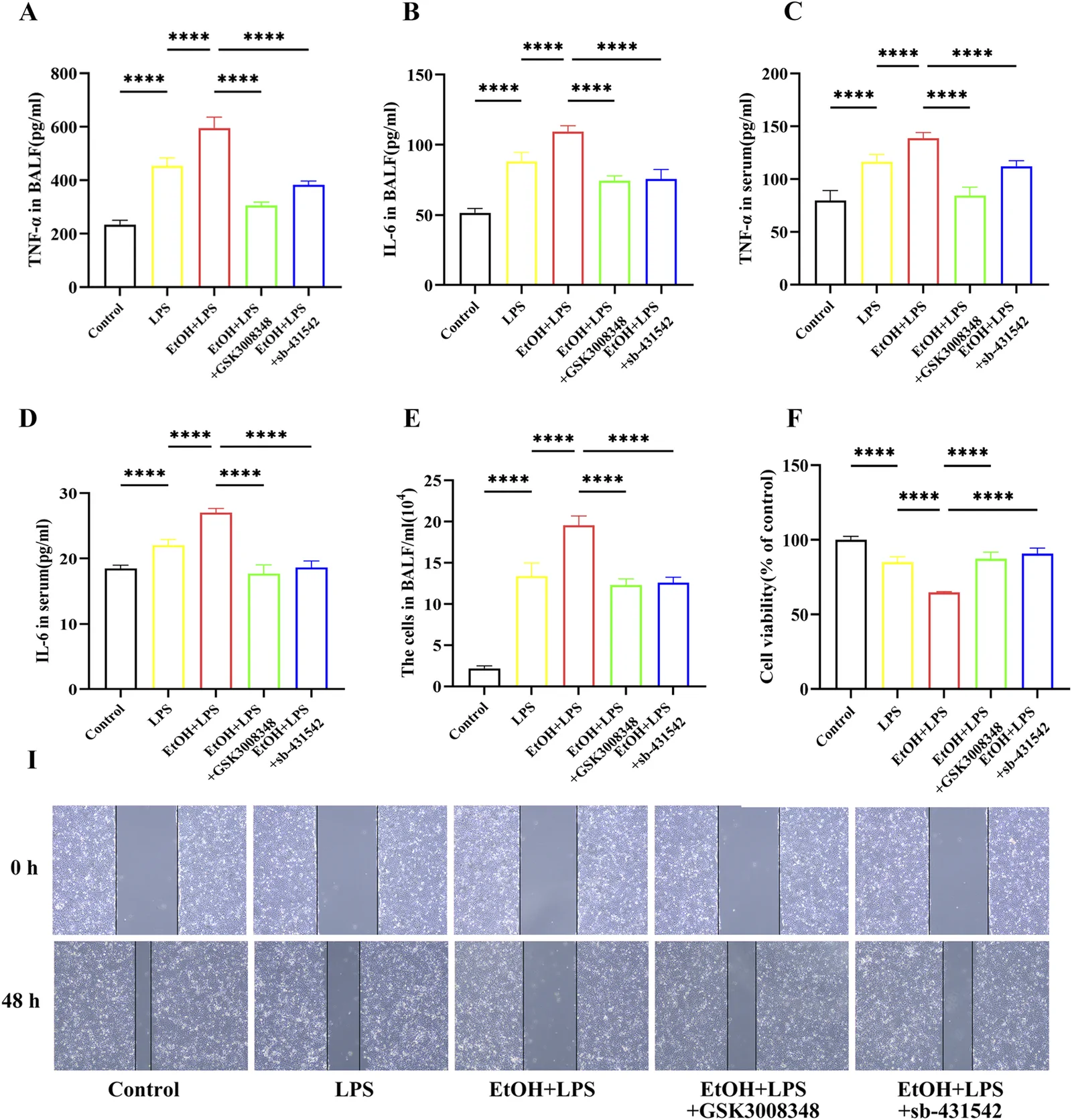
The Integrin αvβ6/TGF-β1/Smad4 pathway mediates chronic alcohol-induced ARDS, thereby exacerbating pulmonary inflammation in mice. *In vitro* studies revealed that activation of this pathway reduced cell viability, whereas its inhibition alleviated these effects. BALF concentrations of TNF-α **(A)** and IL-6 **(B)**. Serum concentrations of TNF-α **(C)** and IL-6 **(D)**. **(E)** Total cell count in BALF. **(F)** Cell viability assessed by CCK-8 assay. **(G)** Wound healing assay images at 0 h and 48 h (×40).Data are presented as mean ± SD (n = 3), with significance levels indicated as *P < 0.05,**P < 0.01, ***P < 0.001, and****P < 0.0001.

### 3.4 Chronic alcohol exposure activates the integrin αvβ6/TGF-β1/Smad4 pathway

Chronic alcohol abuse has been shown to activate TGF-β1, contributing to pulmonary injury (Bechara et al., 2004). TGF-β1 activation in alveolar epithelial cells depends on Integrin αvβ6 (Kim et al., 2018). In both *in vivo* (Figures 4A–C) and *in vitro* (Figures 4D–F) models, chronic alcohol exposure-induced ARDS triggers activation of this signaling pathway, accompanied by increased expression of the Integrin αvβ6/TGF-β1/Smad4 pathway. Shown in Figure 4, compared with the control group, inhibition of Integrin αvβ6 significantly reduced the alcohol-induced activation of this signaling cascade. Inhibition of TGF-β1 also diminished the role of Integrin αvβ6 in pathway activation.These experimental results demonstrate that the Integrin αvβ6/TGF-β1/Smad4 pathway is activated in alcohol-induced ARDS and further confirm the upstream-downstream relationship of this pathway.​

**FIGURE 4:**
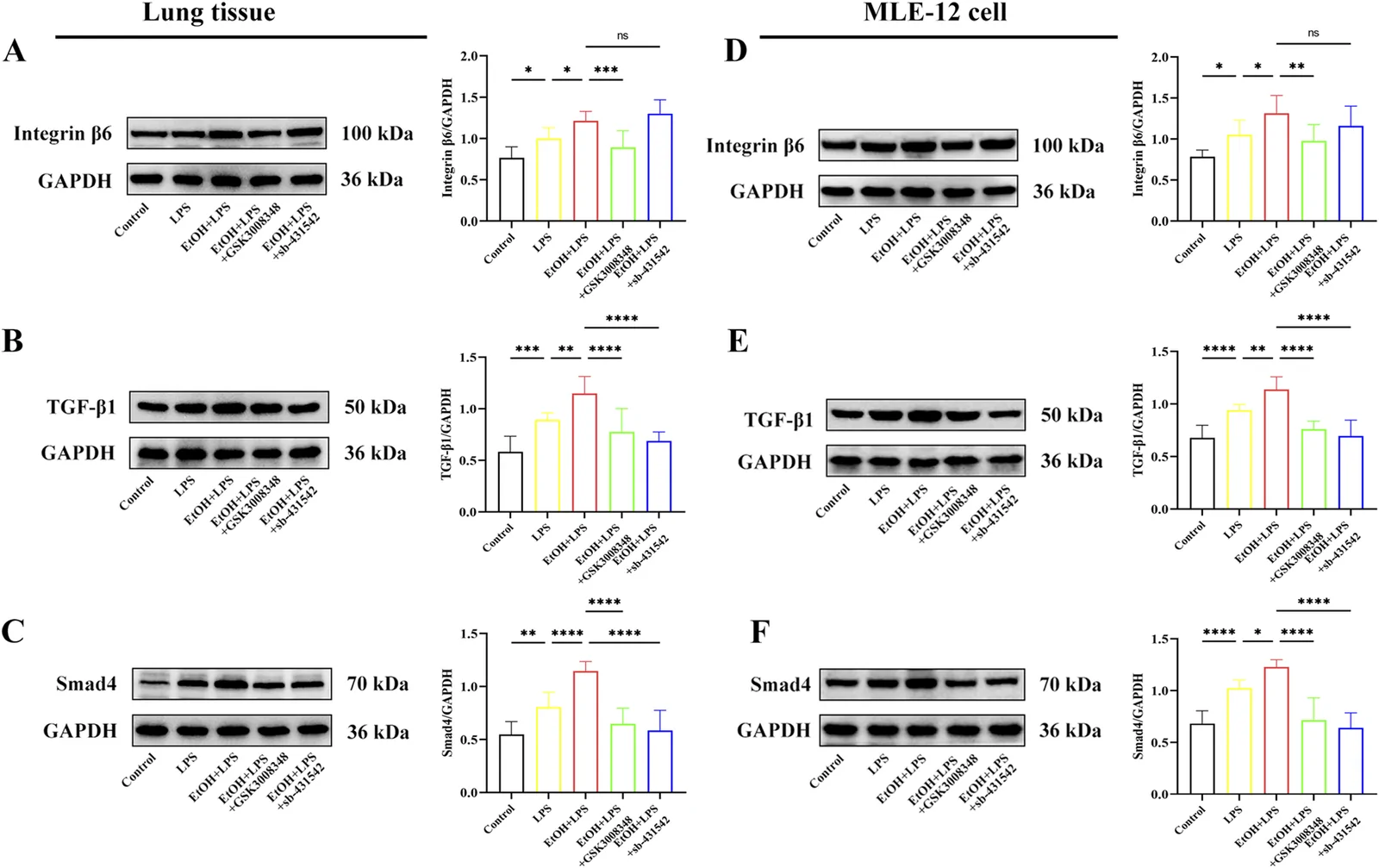
Chronic alcohol exposure impairs alveolar fluid clearance via the Integrin αvβ6/TGF-β1/Smad4 pathway. Western blot analysis was used to detect the protein expression of Integrin αvβ6 **(A)**, TGF-β1 **(B)**, and Smad4 **(C)** in lung tissue.Western blot analysis revealed the protein levels of Integrin αvβ6 **(D)**, TGF-β1 **(E)**, and Smad4 **(F)** in MLE-12 cells.Data are presented as mean ± SD (n = 3), with significance levels indicated as *P < 0.05,**P < 0.01, ***P < 0.001, and****P < 0.0001.

### 3.5 Role of Smad4 in sodium transporter regulation in alcohol-induced ARDS

Previous studies have shown that Smad4 regulates sodium transporters in the kidney and heart, but its role in pulmonary sodium transporters during alcohol-induced ARDS remains unclear. In alveolar epithelial cells pretreated with alcohol and subsequently exposed to LPS, the expression of sodium transporters was reduced compared to LPS treatment alone (Figures 5B,C). After confirming effective Smad4 knockdown (Figure 5A), Smad4 knockdown attenuated the alcohol-induced suppression of ENaC-α (Figure 5B) and Na^+^,K^+^-ATPaseα1 (Figure 5C) expression.

**FIGURE 5:**
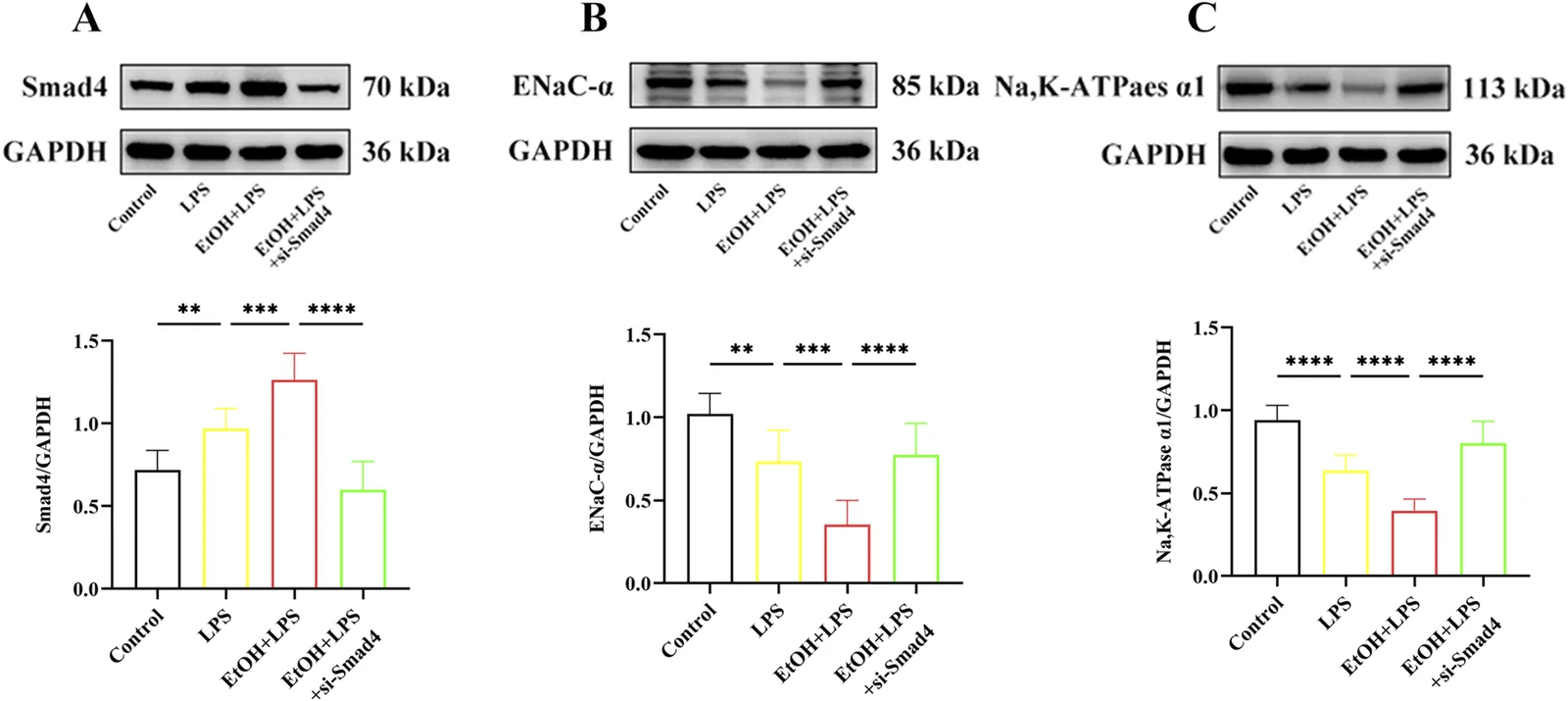
Chronic alcohol-induced ARDS is mediated by Smad4-targeted downregulation of ENaC and Na^+^,K^+^-ATPase expression. **(A)** Western blot analysis of Smad4 protein expression in each group. Western blot analysis of ENaC - α1 **(B)** and Na^+^,K^+^-ATPase α1 **(C)** protein expression in each group.Data are presented as mean ± SD (n = 3), with significance levels indicated as *P < 0.05,**P < 0.01, ***P < 0.001, and****P < 0.0001.

### 3.6 Chronic alcohol exposure regulates sodium transporters via the integrin αvβ6/TGF-β1/Smad4 pathway

Alveolar epithelial cells play a crucial role in maintaining fluid balance for optimal gas exchange. ENaC and Na^+^,K^+^-ATPase are essential for clearing pulmonary edema (Wynne et al., 2017) (Nong et al., 2022). These channels maintain a relatively dry alveolar environment by actively transporting sodium. ENaC consists of three subunits (α, β, γ), while Na^+^,K^+^-ATPase comprises two subunits (α1, β1). The expression levels of these subunits were assessed to evaluate pulmonary fluid clearance. WB analysis in both *in vivo* and *in vitro* models revealed that the alcohol - induced ARDS group showed a significant decrease in the expression of ENaC subunits (Figures 6A,B) and Na^+^,K^+^-ATPase (Figures 7A,B) compared to the ARDS group without alcohol induction.However, inhibition of the Integrin αvβ6/TGF-β1 pathway restored their expression levels.

**FIGURE 6:**
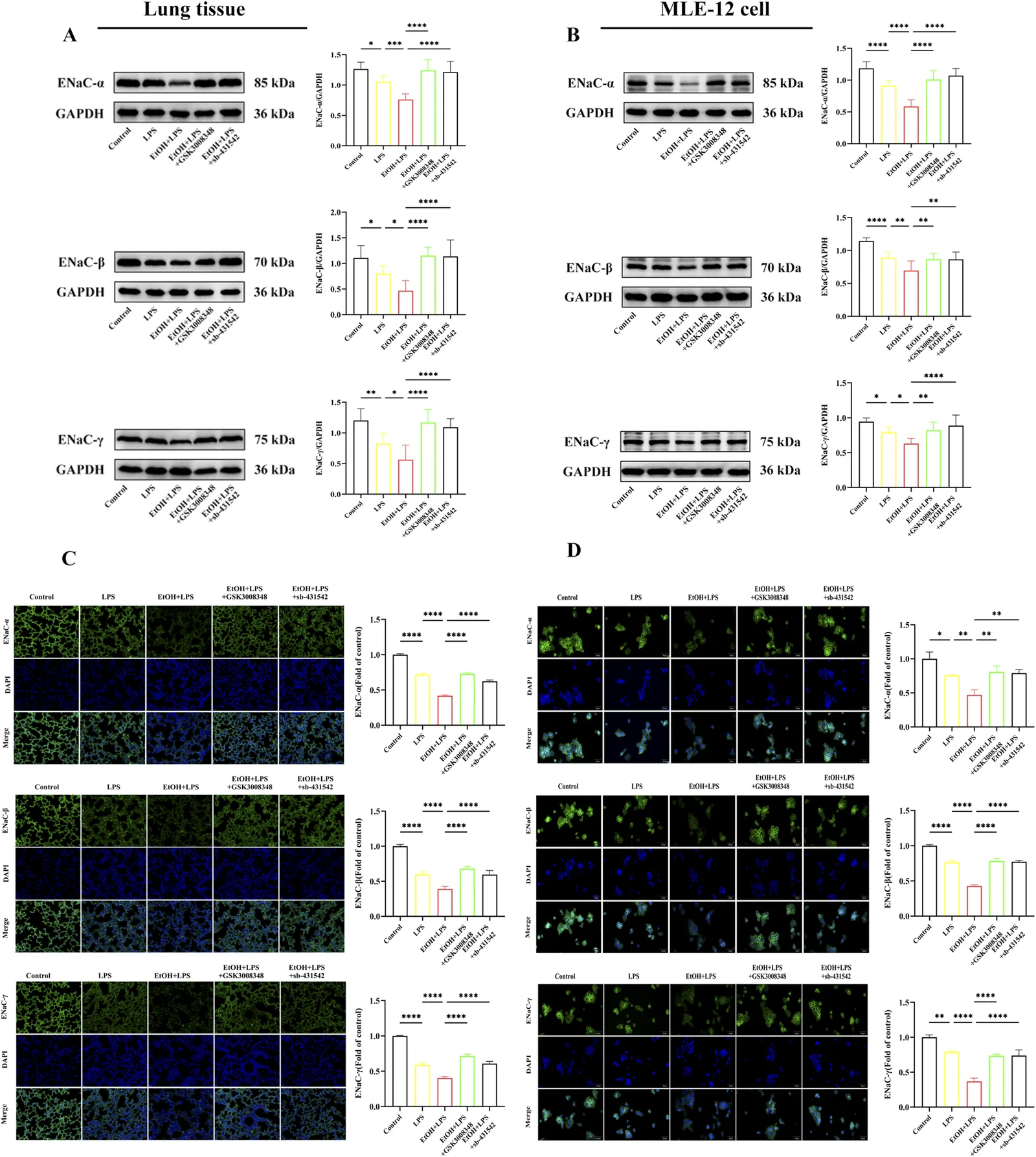
The Integrin αvβ6/TGF-β1/Smad4 pathway mediates the localization and expression of ENaC subunits in mouse lung tissue and MLE-12 cells under chronic alcohol-induced ARDS. Western blot analysis was performed to determine the protein expression of ENaC subunits **(A)** in lung tissues.Western blot analysis quantified ENaC subunits **(B)** protein expression in MLE-12 cells across experimental groups in cell-based assays. Immunofluorescence analysis of ENaC subunits **(C)** protein expression and localization in mouse lung tissue.Immunofluorescence analysis of ENaC subunits **(D)** protein expression and localization in MLE-12 cells. Data are presented as mean ± SD (n = 3), with significance levels indicated as *P < 0.05,**P < 0.01, ***P < 0.001, and****P < 0.0001.

**FIGURE 7:**
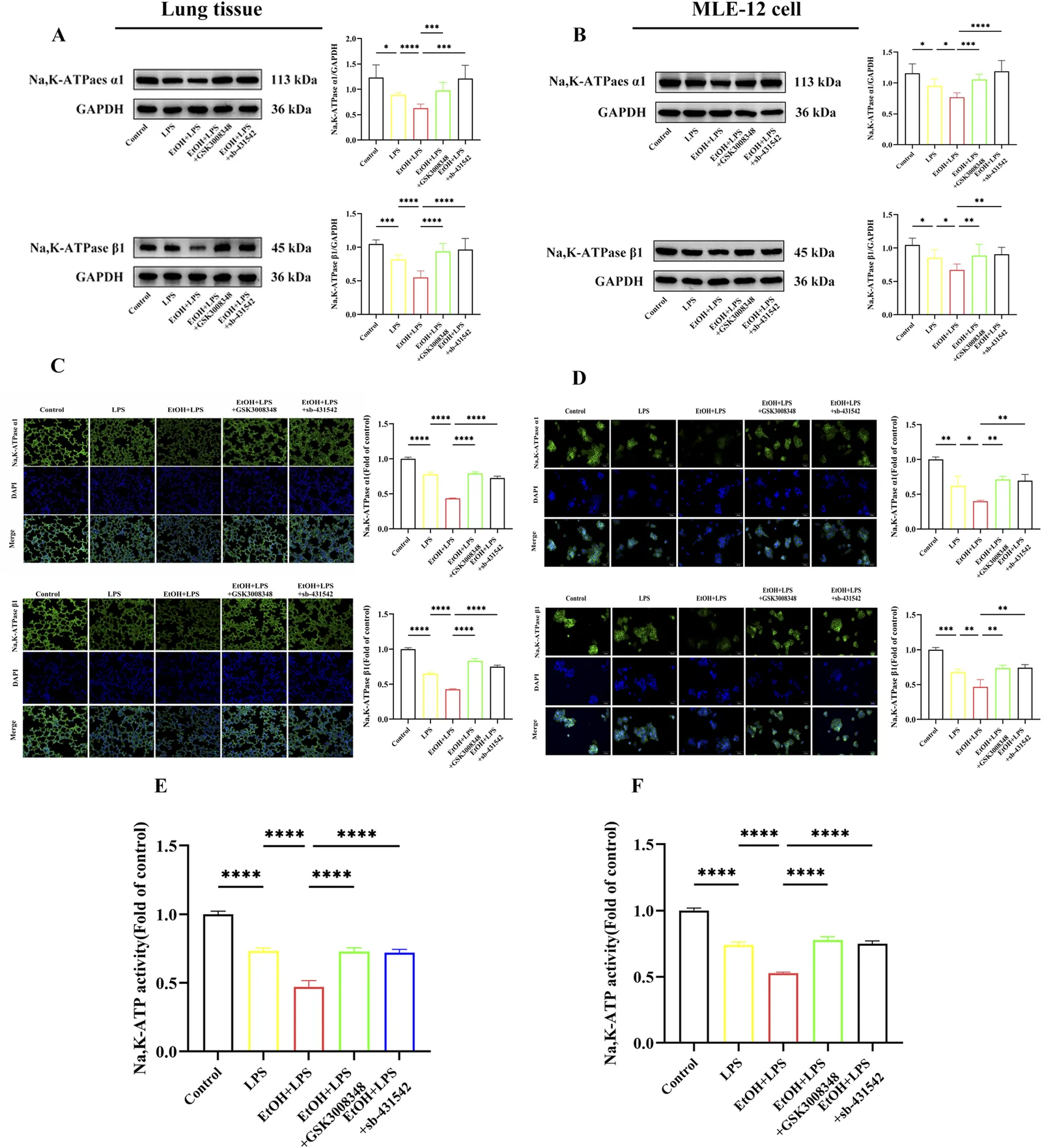
To investigate the regulatory effect of the Integrin αvβ6/TGF-β1/Smad4 pathway on Na^+^,K^+^-ATPase subunits expression and distribution in chronic alcohol-induced ARDS, we examined Na^+^,K^+^-ATPase subunits levels in both mouse lung tissue and MLE-12 cells. WB revealed marked changes in Na^+^,K^+^-ATPase subunits protein abundance in lung tissue following chronic alcohol exposure and LPS challenge. **(A)** Consistent results were obtained in MLE-12 cells, where the expression of ENaC subunits was significantly altered in the chronic alcohol-induced ARDS group and partially restored upon pathway inhibition. **(B)** Immunofluorescence staining further demonstrated that chronic alcohol-induced ARDS disrupts the membrane-associated localization of ENaC subunits in lung tissue **(C)** and MLE-12 cells **(D)**, an effect that was alleviated by blockade of the Integrin αvβ6/TGF-β1/Smad4 signaling cascade. Na^+^,K^+^-ATPase activity assay in lung tissue **(E)** and MLE-12 cells. **(F)** Data are presented as mean ± SD (n = 3), with significance levels indicated as *P < 0.05,**P < 0.01, ***P < 0.001, and****P < 0.0001.

Immunofluorescence results showed that chronic alcohol exposure-induced ARDS significantly reduced the fluorescence signal intensity of ENaC (Figures 6C,D) and Na^+^,K^+^-ATPase (Figures 7C,D) whereas inhibition of the integrin αvβ6/TGF-β1/Smad4 pathway markedly restored their fluorescence signals in the alcohol-treated group. Both *in vivo* (Figure 7E) and *in vitro* (Figure 7F) studies demonstrated that chronic alcohol consumption inhibits Na^+^,K^+^-ATPase activity, while suppression of the Integrin αvβ6/TGF-β1 pathway resulted in partial recovery of its function.These findings indicate that the Integrin αvβ6/TGF-β1/Smad4 axis plays a regulatory role in ENaC and Na^+^,K^+^-ATPase expression.

## 4 Discussion

ARDS is associated with high morbidity and mortality in both the short and long term (Kim et al., 2018). The pathogenesis of ARDS is complex, and anti-inflammatory therapy is a potential strategy to improve outcomes. It is characterized by the accumulation of protein-rich pulmonary edema, surfactant dysfunction, and impaired gas exchange, all contributing to severe clinical outcomes (Huppert and Matthay, 2017). Ethanol exposure increases oxidative stress in the lungs, thereby aggravating alveolar epithelial injury and pulmonary inflammation (Guidot and Roman, 2002). Excessive alcohol consumption increases the risk of lung diseases (Malherbe and Messaoudi, 2022), exacerbates pulmonary edema, and impairs its clearance (Deng et al., 2021), though the precise mechanisms remain unclear. Our study demonstrates that chronic alcohol-induced ARDS is associated with more severe pulmonary edema and inflammation, along with reduced pulmonary fluid clearance. One of the key mechanisms involved is the Integrin αvβ6/TGF-β1/Smad4 signaling pathway. Chronic alcohol abuse appears to worsen lung injury by activating the Integrin αvβ6/TGF-β1/Smad4 pathway.

ENaC plays a vital role in AFC and systemic fluid homeostasis by mediating sodium absorption across the apical membrane of epithelial cells into the interstitial space (Vadász and Sznajder, 2017). Composed of three subunits (α, β, γ), ENaC is expressed in alveolar epithelial cells and serves as a key channel protein promoting AFC. Genetic deletion of ENaC in these cells significantly impairs fluid clearance and increases lung water content (Aufy et al., 2023). The present study primarily focused on validating the molecular mechanisms of the Integrin αvβ6/TGF-β1/Smad4 pathway, thus quantitative analysis was confined to ENaC protein expression levels. Although a well-documented positive correlation exists between ENaC expression and its functional activity (Xin et al., 2025), direct functional evidence would provide more compelling support for this regulatory mechanism, representing a crucial direction for our subsequent research. As a result, ENaC expression are essential in mitigating pulmonary edema. Na^+^ transporters, particularly ENaC and Na^+^,K^+^-ATPase, are central to pulmonary fluid regulation. Na^+^,K^+^-ATPase, located on the basolateral membrane of alveolar epithelial cells, actively transports sodium out of cells and plays a critical role in maintaining alveolar fluid balance (Zhou et al., 2008). While pulmonary edema is a hallmark of ARDS-related hypoxemia, and chronic alcohol consumption exacerbates it, the mechanisms underlying this worsening effect remain incompletely understood.

Chronic alcohol consumption can damage alveolar epithelial cells by upregulating Integrin αvβ6 (Curry-McCoy et al., 2013), in addition, chronic alcohol consumption increases TGF-β1 levels, resulting in alveolar epithelial injury (Bechara et al., 2004). TGF-β1 is involved in both pulmonary edema resolution and sodium channel regulation in renal tissue (Wakida et al., 2006). Chronic alcohol consumption may regulate alveolar epithelial cells through Integrin αvβ6. In the lungs, TGF-β1 modulates pulmonary fibrosis through downstream Smads (Cui et al., 2021). While its role in pulmonary fibrosis is well established, its contribution to alcohol-induced ARDS is less understood.In cardiac tissue, TGF-β1/Smad4 signaling is known to regulate sodium and potassium channels (Pang et al., 2024). However, whether chronic alcohol-induced ARDS affects sodium channels via Smad4 was previously unknown. Our results demonstrate that chronic alcohol exposure leads to increased Smad4 expression and altered expression of sodium transporters. *In vitro*, Smad4 knockdown mitigated alcohol-induced suppression of sodium transporter expression, suggesting that Smad4 negatively regulates these transporters and contributes to edema in alcohol-induced ARDS.

Our study found that inhibiting TGF-β1 prevented alcohol-induced Smad4 activation, confirming that Smad4 acts downstream of TGF-β1,and Integrin αvβ6 plays a crucial upstream role in the pathogenesis of alcohol-induced ARDS.Integrin αvβ6 enhances TGF-β1 activation, which promotes lung injury.Integrin αvβ6 is critical in amplifying this signaling cascade. Our findings show that Integrin αvβ6 and TGF-β1 expression are elevated in ARDS, especially in alcohol-induced cases. Notably, blocking Integrin αvβ6 prevents alcohol-driven TGF-β1 elevation; however, TGF-β1 inhibition does not alter Integrin αvβ6 levels in alcohol-induced ARDS, confirming that TGF-β1 functions downstream of Integrin αvβ6. These findings reveal that Integrin αvβ6 mediates the TGF-β1/Smad4 signaling cascade in regulating sodium transporter expression in alcohol-induced ARDS.

One of this study’s limitation is that it focused primarily on Smad4 as the downstream effector of TGF-β1; therefore, the roles of other downstream targets require further exploration. Additionally, we relied on MLE-12 cells without validating findings in primary alveolar epithelial cells; future studies should include multiple cell types. Finally, the findings are based on murine models and *in vitro* experiments, and their relevance to human physiology needs further investigation.

## 5 Conclusion

In summary, our study reveals that chronic alcohol exposure exacerbates pulmonary edema in ARDS by inhibiting sodium channels via the Integrin αvβ6/TGF-β1/Smad4 signaling pathway. These findings identify this axis as a key mediator of alcohol-induced ARDS and offer novel molecular insights into its pathogenesis.

## Data Availability

The original contributions presented in the study are included in the article/supplementary material, further inquiries can be directed to the corresponding author.
